# The impact of disability, fatigue and sleep quality on the quality of life in multiple sclerosis

**DOI:** 10.4103/0972-2327.44559

**Published:** 2008

**Authors:** Haleh Ghaem, Afshin Borhani Haghighi

**Affiliations:** Department of Epidemiology, School of Health and Nutrition, Shiraz University of Medical Sciences, Shiraz, Iran; 1 Department of Neurology, Nemazi Hospital, Shiraz University of Medical Sciences, Shiraz, Iran

**Keywords:** Disability, fatigue, multiple sclerosis, quality of life, sleep quality

## Abstract

**Background:**

Only few papers have investigated the impact of multiple sclerosis (MS), especially MS-related fatigue and the impact of the quality of sleep on the quality of life (QoL) in MS patients.

**Objective:**

The objective of this study was to measure the quality of life in MS patients and the impact of disability, fatigue and sleep quality, using statistical modeling.

**Materials and Methods:**

A cross-sectional study was conducted and data was collected from 141 MS patients, who were referred to the Mottahari Clinic, Shiraz, Iran, in 2005. Data on health-related quality of life (MSQoL-54), fatigue severity scale (FSS), and Pittsburgh sleep quality Index (PSQI) were obtained in the case of all the patients. Epidemiology data concerning MS type, MS functional system score, expanded disability status scale (EDSS) etc. were also provided by a qualified neurologist. Spearman α coefficient, Mann-Whitney U test, and linear regression model were used to analyze the data.

**Results:**

The mean ±SD age of 141 MS patients was 32.6±9.6 year. Thirty five (24.8%) of them were male and the others were female. Eighty two (58.1%) of the patients had EDSS score of ≤ 2, 36 (25.5%) between 2.5 and 4.5, and 23 (16.3%) ≥ 5. As per PSQI scores, two (1.4%) of the patients had good sleep, 16 (11.3%) had moderate sleep and 123 (87.2%) had poor sleep. There was a significant high positive correlation between the quality of mental and physical health composite scores (r = 0.791, *P*<0.001). There was a significant negative correlation between the quality of physical score and age (r = -0.88, *P*<0.001), fatigue score (r = -0.640, *P*<0.001), EDSS score (r = -0.476, *P*<0.001) and PSQI (sleep quality r = -0.514, *P*<0.000). Linear regression analysis showed that PSQI score, EDSS, and fatigue score were predictors in the model between the quality of physical score and covariates (*P*<0.001). Linear regression model showed that fatigue score and PSQI were predictors in the model between the quality of mental score and covariates (*P*<0.001).

**Discussion and Conclusion:**

In conclusion, it may be said that MS patients had poor and moderate quality of mental and physical health. The quality of life was impaired as seen by PSQI, EDSS, and FSS. It is our suggestion that these patients require the attention of health care professionals, to be observed for the need of possible psychological support.

## Introduction

Multiple sclerosis (MS) is one of the most common cause of neurological disability in young adults in several countries. MS has a major impact on the lives of patients. The psychological impact of the disease was found to be significantly associated with the severity of the disability.[1–7]

In the past, only a few authors have investigated the influence of MS-related fatigue on QoL. The expanded disability status scale (EDSS)[1] is the most common measure of impairment and disability for MS patients and outcome in clinical trials. Most studies showed that the disability status had a limited influence on QoL[8–11] and some studies showed that QoL was correlated with disability,[12–14] whereas fatigue[15–19] was clearly associated with reduced QoL scores in patients with multiple sclerosis. Only one study assessed the impact of fatigue and other determinants on QoL, using a linear regression model.[15] There is only one study that measured the impact of sleep quality on QoL.[12]

Fatigue is one of the three most frequently disabling symptoms of MS[20] and may be considered abnormal in as many as 78% of the patients.[21] It is severe enough to prevent a patient from carrying out his or her duties and responsibilities or to interfere with work, family life, and social life.[22] The prevalence of sleep complaints was three times greater in a group of MS patients than in controls.[23]

As only one study has considered the impact of sleep quality, fatigue, disability and demographic data on QoL in MS patients using statistical modeling up till now, this study deals with the assessment of QOL in MS using the MSQoL-54, a disease specific instrument, and with the analysis of its determinants in a clinical series of subjects with multiple sclerosis, using statistical modeling.

## Materials and Methods

### Patients

The subjects were consecutive patients (both newly diagnosed and follow-up), who were referred to the MS clinic at the Nemazee Hospital in Shiraz, south of Iran, from June 2005 to December 2005.

The inclusion criteria were clinically definite or laboratory supported MS, according to Poser criteria.

Demographic data like age, sex, marital status, socio-economic status, and education were recorded. Clinical data concerning MS type, duration of the disease, functional system score and expanded disability status scale (EDSS) of the patients were also provided by a qualified neurologist. All the patients signed the informed consent.

Literate patients filled out the questionnaire by themselves. In the case of illiterate patients, the questionnaire was filled out by unbiased test operators, with the help of verbal communications.

### Instruments

#### A. MSQoL-54

The SF-36 questionnaire is one of the most widely used health related quality of life (HRQoL) instruments in the United States. It was devised to satisfy the minimum psychometric standards necessary for group comparisons involving general health dimensions (not specific to age, disease, or treatment group).[24] An additional question was also used, which asked about self-evaluated change in health status. The MS-18 module, originally devised in the United States in 1995, adds 18 additional items concerned with the following areas: health distress, sexual function, satisfaction with sexual function, overall quality of life, cognitive function, and energy, to SF-36. The composite instrument, composed of SF-36 and MS-18, is MSQOL-54 which contains 52 items grouped into 12 scales, plus two lone items.[25]

*Pittsburgh Sleep Quality Index*: The Pittsburgh Sleep Quality Index (PSQI) is an effective instrument used to measure the quality and patterns of sleep in older adults. It differentiates ‘poor’ from ‘good’ sleep by measuring seven areas: subjective sleep quality, sleep latency, sleep duration, habitual sleep efficiency, sleep disturbances, use of sleeping medication, and daytime dysfunction over the last month.[26]

#### B. Fatigue Severity Scale

The Fatigue Severity Scale (FSS) is a method of evaluating fatigue in multiple sclerosis and other conditions including Chronic Fatigue Immune Dysfunction Syndrome (CFIDS) and Systemic Lupus Erythmatosis (SLE). The subject is asked to read each statement and circle a number from 1 to 7, depending on how appropriate he/she felt the statement applied to him/her over the preceding week. A low value indicates that the statement is not very appropriate whereas a high value indicates agreement.[27]

Internal consistency reliability in this study was good.

Coefficient α was 0.96 for MSQoL- 54; 0.92 for PSQI; and 0.89 for FSS.

### Statistical analysis

Continuous data were given as a mean. Categorical data were given as counts and percentages. Spearman correlation coefficient was used to test if there was any correlation between quality of mental and physical scores and age, duration of disease, fatigue severity, EDSS, and sleep quality. The linear regression model was used for statistical modeling on the quality of life and covariates. In order to measure the impact of covariates on QoL, we included all covariates (PSQI, EDSS, and fatigue scores, age, years of education, sex, marital status, and duration of disease), with quality of mental and health composite as a dependent variable in a model.

In order to test for any association between quality of life and sex, Mann-Whitney U was used.

To check for any association between quality of life and MS type, Kruskal-Wallis test was used.

If the observations were made on variables x (covariates) and y (dependent variable) for a large number of individuals, we were interested in the way in which y changes on the average, as x assumed different values. If it was appropriate to think of y as a random variable for any given value of x, we could enquire how the expectation of y changed with x. The probability distribution of y when x is known is referred to as conditional distribution.[28]

Statistical Package for Social Sciences (SPSS) 13.0 was used to analyze the data.

## Results

### A: The demographics and the characteristics of MS

The general characteristics of the 141 MS patients included: age range (16-60 years) with mean ± SD 32.2±9.7 years. Out of them, 35 (24.8%) were male and 106 (75.2%) were female patients. Fifty three (37.6%) of the patients were single, 80 (56.7%) married and seven (5%) divorced [Table 1]. The mean ± SD age of the onset of MS (time of diagnosis by medical professionals) was 28.9±8.8 years. The duration of MS disease in 71 (50.4%) of the patients was ≤ one year; between two and four years in 34 (24.1%) of them and ≥ five years in 36 (25.5%) of them [Table 1].

**Table 1:** Frequency and percentage of demographic and clinical data in all patients

Variable	Frequency	%
Gender		
Male	35	24.8
Female	106	75.2
Marital status		
Married	80	56.7
Single	53	37.6
Divorced	7	5.0
MS type		
relapsing-remitting	105	74.5
primary progressive	4	2.8
secondary progressive	28	19.9
relapsing progressive	4	2.8
Duration of MS (year)		
≤ 1	71	50.4
2-4	34	24.1
≥ 5	36	25.5
Functional system		
Pyramidal	86	61.0
Brian stem	37	26.2
Cerebellar	47	33.3
Sensory	84	59.6
Bowel and bladder	30	21.3
Cerebral	30	21.3
Visual	60	46.8
EDSS		
≤ 2	82	58.1
2.5-5	36	25.5
≥ 5	23	16.3
PSQI		
Good	2	1.4
Moderate	16	11.3
Poor	123	87.2
Fatigue		
≤ 36	76	53.9
> 36 (suffering from fatigue)	65	46.1

The MS form of the patients was relapsing - remitting in 105 (74.5%), primary progressive in four (2.8%), secondary progressive in 28 (19.9%), and relapsing - progressive in four (2.8%) [Table 1].

The functional system of the patients was pyramidal in 86 (61%), brain stem in 37 (26.2%), cerebellar in 47 (33.3%), sensory in 84 (59.6), bowel and bladder in 30 (21.3%), cerebral in 30 (21.3%),and visual in 60 (46.8%) [Table 1].

Eighty two (58.1%) of the patients had EDSS score ≤ 2, 36 (25.5%) between 2.5 and 4.5 and 23 (16.3%) ≥ 5. According to PSQI scores, two (1.4%) patients had good sleep, 16 (11.3%) of them moderate sleep and 123 (87.2%) of them had poor sleep [Table 1]. The mean ± SD PSQI was 9.5± 3.7, FSS was 4.5±1.8 and EDSS 2.3±2.1.

Descriptive statistics of the 141 MS patients is shown in Table 2.

**Table 2:** Descriptive statistics of quality of life, fatigue, sleep quality, EDSS, and age in all patients

Variable	N	Median	Mean	SD*	95% CI**
Health	141	50.0	47.9	34.0	42.3-53.5
Satisfaction with sexual function	83	75.0	61.1	33.7	53.9-68.3
Physical function	141	65.0	57.5	32.1	52.2-62.8
Role limitation due to physical problems	141	25.0	39.9	40.5	33.2-46.6
Role limitation due to emotional problems	141	33.3	46.1	42.7	39.05-53.15
Pain	141	65.0	64.9	26.9	60.5-69.3
Emotional well-being	141	48.0	48.2	22.1	44.6-51.8
Energy	141	40.0	42.4	20.7	39.0-45.8
Health perceptions	141	50.0	50.3	22.4	46.6-54.0
Social function	141	66.7	67.1	24.6	63.0-71.2
Cognitive function	141	75.0	67.4	28.9	62.7-72.1
Health distress	141	60.0	58.4	28.4	53.7-63.1
Sexual function	83	83.3	68.3	33.6	61.1-75.5
Overall quality life	141	55.0	57.1	24.1	53.2-61.0
Physical health composite	141	49.5	52.9	21.4	49.4-56.4
Mental health composite	141	53.1	53.6	22.6	49.9-57.3
Age	141	30.0	32.2	9.7	30.6-33.8
Sleep quality	141	9.0	9.5	3.7	8.9-10.1
Fatigue severity	141	34.0	31.98	12.89	29.88-34.08
EDSS	141	2.0	2.3	2.1	1.95-2.65
Diagnosis age of MS	141	28.0	28.9	8.8	27.5-30.3

* SD: standard deviation

** CI: confidence interval

### B: Correlations

There was a significant high positive correlation between the quality of mental and physical health composite scores of MS patients (r = 0.791, *P*<0.001). There was a significant negative correlation between quality of physical score and age (r = -0.88, *P* <0.001), fatigue score (r = -0.640, *P* < 0.001), EDSS score (r = -0.476, *P* <0.001) and PSQI (sleep quality r = -0.514, *P* <0.001) [Table 3].

**Table 3:** Correlation between physical health composite, mental health composite, fatigue, sleep quality, age and EDSS in all patients

Variable		Mental	Physical	Fatigue	Sleep	EDSS
Physical	Correlation coefficient	0.791	-	-0.640	-0.514	-0.476
	Sig (2 tailed)	0.001		0.001	0.001	0.001
Mental	Correlation coefficient	-	-	-0.599	-0.514	-0.273
	Sig (2 tailed)			0.001	0.001	0.004
Fatigue	Correlation coefficient	-	-	-	0.473	0.350
	Sig (2 tailed)				0.001	0.001
Sleep	Correlation coefficient	-	-	-	-	0.138
	Sig (2 tailed)					0.156
Age	Correlation coefficient	-0.186	-0.880	0.268	0.241	0.332
	Sig (2 tailed)	0.028	0.002	0.001	0.004	0.001

There was a significant negative correlation between the quality of mental score and age (r = -0.186, *P* =0.028), fatigue score (r = -0.599, *P* <0.001), EDSS score (r = -0.273, *P*=0.004) and PSQI score (sleep) (r = -0.514, *P*<0.001) [Table 3].

There was a significant correlation between fatigue and sleep quality (r = 0.473, *P*<0.001), fatigue and EDSS (r = .350, *P* < 0.001), age and fatigue (r = 0.268, *P* < 0.001), age and sleep (r = .241, *P* =0.004) and age and EDSS ( r= .332, *P* <0.001)) [Table 3].

There was a significant association between the quality of physical score and MS type (*P* = 0.010). The MS patients who were relapsing-remitting and relapsing-progressive had better quality physical health than those who were primary-progressive and secondary progressive [Table 4].

**Table 4:** Association between the quality of physical and mental health, according to the form of MS

Form of MS		Physical health	Mental health
Relapsing-remitting	Number	105	105
	Mean±SD	56.8±18.9	55.3±21.3
Primary progressive	Number	4	4
	Mean±SD	42.6±14.6	59.3±37.1
Secondary progressive	Number	28	28
	Mean±SD	37.1±16.6	45.3±22.7
Relapsing progressive	Number	4	4
	Mean±SD	52.7±19.8	53.6±21.9
*P* value		0.010	0.349

There were no significant differences between the quality of mental score and sex (*P* = 0.642) and also the quality of physical score and sex (*P* = 0.310). No significant association between the quality of mental score and MS type was seen (*P* = 0.349) [Table 4].

### C: The results of the outcome measures

In order to measure the impact of covariates on QOL, we included all covariates (PSQI, EDSS, and fatigue scores, age, years of education, sex, marital status, duration of disease) with the quality of mental and health composite as a dependent variable in a model. The linear regression analysis showed that PSQI, EDSS, and fatigue scores were predictors in the model between the quality of physical score and covariates (*P* <0.001).

Linear regression analysis also showed that fatigue and PSQI scores were predictors in the model between the quality of mental scores and covariates (*P* <0.001).

There was no significant correlation between the quality of mental and physical health scores and duration of disease, and years of education.

## Discussion

We used MSQoL-54, which includes additional questions about interpersonal function, social, emotional, personal and spiritual fulfillment. We used the instrument (MSQoL-54) that had been used by other researchers.[15,17,29] This questionnaire was translated and validated in Italian,[8] French,[30,31] French Canadian[32] and Turkish[33] and Farsi Language.[34] Besides, we considered measures of fatigue severity, sleep quality, a comprehensive insight into the patient's life, their disabilities, impairments and handicap and attempted to determine the impact of these factors on the quality of life.

There are few studies on the quality of life and impact of fatigue and sleep together in MS patients.[12]

In our study, generally, the patients scored middle and low on all QoL subscales. Benedict *et al*., in their studies, reported that HRQoL was poor in MS patients.[17]

Previous studies demonstrated that psychological well-being and quality of life were reduced in MS patients and were inversely related to the disability status.[19,25,35–40]

Our study showed a major impact of the severity of disease on the quality of both physical and mental health in MS patients. Patients who had upper scores on EDSS, reported lower scores on the quality of physical and mental health. This finding was supported by other studies.[12–14] Merkerbach *et al*. showed that only physical health composite inversely related to EDSS.[19,29] The quality of mental and physical health were correlated with each other.

Our finding showed that the patients who were older had lower quality of mental and physical health than younger ones. In the study by Merkerbach *et al*., only the physical health composite related inversely to age.[19]

However, this finding was not supported by the linear regression model. Therefore, this impact of age occurred perhaps due to the association between age and severity of the disease.

The current study showed that patients who had upper scores in fatigue severity and sleep quality, had lower scores in the quality of physical and mental health composite. This finding is supported by some other researchers.[12,15,16,18,19,41]

Our study showed a positive correlation between EDSS and fatigue, that had been shown by other studies done earlier.[14,16]

Sex, marital status, duration of the disease, and years of education had no impact on the quality of life. Only one study showed that a correlation between the duration of disease and physical health.[19]

Linear regression analysis showed that PSQI, EDSS, and fatigue scores were predictors in the model between the quality of physical score and covariates (*P*<0.001). Linear regression analysis also showed that fatigue and PSQI scores were predictors in the model between the quality of mental scores and covariates (*P*<0.001).

As there was only six illiterate patients in this study, we could not compare the results according to literacy. So, we suggest that other researchers consider it. As far as we know, there are a few studies with similar methodology in medical studies. Our finding was supported by only the study that used linear regression.[12]

In conclusion, this study demonstrated that MS patients had poor and moderate quality of mental and physical health. The EDSS score, severity of fatigue and sleep quality were significant indicators that correlated with the quality of physical and mental health. This study also showed that demographic data (age, sex, marital status, years of education) and duration of disease had no effect on QoL, after conducting statistical modeling. As the quality of mental health and that of physical health have a high relationship with each other, we suggest that MS patients require the attention of health care professionals to observe those who may need further psychological support As the fatigue and sleep problems disturbed the quality of life, we suggest earlier and more effective treatment of these aspects of the MS patients. Patients should organize their activities to permit rest periods when needed. Highly demanding activities should be scheduled for times of the day when fatigue is less likely. Also, ways to improve sleep hygiene should be imparted to MS patients.
